# A Low-Noble-Metal Ru@CoMn_2_O_4_ Spinel Catalyst for the Efficient Oxidation of Propane

**DOI:** 10.3390/molecules29102255

**Published:** 2024-05-11

**Authors:** Yan Cui, Zequan Zeng, Yaqin Hou, Shuang Ma, Wenzhong Shen, Zhanggen Huang

**Affiliations:** 1 State Key Laboratory of Coal Conversion, Institute of Coal Chemistry, Chinese Academy of Sciences, Taiyuan 030001, China; cuiyan@sxicc.ac.cn (Y.C.); zengzequan@sxicc.ac.cn (Z.Z.); houyaqin@sxicc.ac.cn (Y.H.); mashuang20@mails.ucas.ac.cn (S.M.); 2 University of Chinese Academy of Sciences, Beijing 100049, China

**Keywords:** Ru@CoMn_2_O_4_, light alkanes, lattice oxygen, oxidation mechanism

## Abstract

Noble metals have become a research hotspot for the oxidation of light alkanes due to their low ignition temperature and easy activation of C-H; however, sintering and a high price limit their industrial applications. The preparation of effective and low-noble-metal catalysts still presents profound challenges. Herein, we describe how a Ru@CoMn_2_O_4_ spinel catalyst was synthesized via Ru in situ doping to promote the activity of propane oxidation. Ru@CoMn_2_O_4_ exhibited much higher catalytic activity than CoMn_2_O_4_, achieving 90% propane conversion at 217 °C. H_2_-TPR, O_2_-TPD, and XPS were used to evaluate the catalyst adsorption/lattice oxygen activity and the adsorption and catalytic oxidation capacity of propane. It could be concluded that Ru promoted synergistic interactions between cobalt and manganese, leading to electron transfer from the highly electronegative Ru to Co^2+^ and Mn^3+^. Compared with CoMn_2_O_4_, 0.1% Ru@CoMn_2_O_4_, with a higher quantity of lattice oxygen and oxygen mobility, possessed a stronger capability of reducibility, which was the main reason for the significant increase in the activity of Ru@CoMn_2_O_4_. In addition, intermediates of the reaction between adsorbed propane and lattice oxygen on the catalyst were monitored by in situ DRIFTS. This work highlights a new strategy for the design of a low-noble-metal catalyst for the efficient oxidation of propane.

## 1. Introduction

The exhaust emission of petrochemical and coal chemical industries, especially oil storage and transportation processes, usually contains light alkanes. Propane, as a kind of light alkane, is the most difficult class of volatile organic compounds (VOCs) to catalytically degrade. The total bond energy of the C-H bond of propane is as high as 4016 kJ/mol [1,2,3], much more than that of aromatic and oxygenic hydrocarbons in the field of printing, dyeing, and spraying industries which are easy to catalytically oxidize [4,5,6]. Consequently, the degradation of propane requires high-activity catalysts and high combustion temperatures [7,8,9,10]. Noble-metal catalysts have exhibited excellent performance in the low-temperature catalytic combustion process, wherein the catalysts with Pt and Pd as active components for light alkanes’ combustion have been widely reported [11,12,13,14]. However, the high price of Pt and Pd limit their wide utilization. In contrast, Ru can greatly reduce the production cost due to its low price (approximately half the price), and it has been proved to have high catalytic activity in many chemical reactions [15], such as the ammonia synthesis reaction [16], the olefin complex decomposition reaction [17], organic compound wet oxidation [18], the catalytic combustion of VOCs [19,20], etc. However, noble-metal catalysts easily sinter at high temperature. Improving the utilization rate of noble metals and enhancing stability are key to the development of Ru catalysts.

Transition-metal oxides used as carriers can effectively improve the activity and stability of catalysts. Relying on the structural formula of AB_2_O_4_, spinel oxides can accelerate the electron transfer between transition metals [21] and optimize the electronic structure of the active site [22], thus improving the redox performance. Mn- and Co-based spinel oxides are excellent catalysts for VOC combustion. Benjamin et al. successfully prepared Co-Mn-oxide spinel catalysts for CO oxidation at ambient temperature [23]. It has been reported that high-valence Mn in the cubic phase, including Mn^3+^ and Mn^4+^, facilitated the adsorption and dissociation of oxygen and electron migration, thus showing better oxygen reduction performance. Zhang [24] investigated toluene oxidation over a nanocrystalline Co_3_O_4_ catalyst and found that its unique structural properties gave it a relatively low Co-O bonding energy and a large number of lattice defects. With the advantages of rich valence states and active sites, and controllable composition and structure, spinel oxides are potential noble-metal carriers. In addition, the noble metal embedded in spinel oxides can modulate the electronic structure of the catalyst and promote the orbital hybridization between the noble metal and the carrier, thus affecting its catalytic performance and stability. Ru@NiCo_2_O_4_ has been successfully used in the oxygen evolution reaction, demonstrating that Ru can effectively modulate the metal valence and activity of a spinel [25]. Xiong et al. [26] referenced Pd-Co_2_NiO_4_ spinel catalysts for methane oxidation, and the deposition of PdOx species on the Co_2_NiO_4_ surface moderated the metal–carrier interaction with good stability. However, the Ru-loaded Co-Mn spinel for the catalytic combustion of light alkanes has not been reported before.

In this study, a Co-Mn spinel was used as a carrier to prepare low-noble-metal Ru spinel catalysts with a kind of typical light alkane (propane) to enhance the stability of the noble-metal catalyst. Meanwhile, the addition of the noble metal Ru is expected to increase the electronic fluidity and form rich lattice oxygen and adsorbed oxygen to improve the activity of the catalytic oxidation of propane. The aim is to develop catalysts with high activity, low loading, and good stability for the degradation of propane which meet the practical needs of industrial applications. Meanwhile, XRD, XPS, In situ DRIFT analysis, and other catalyst characterization tools were used to establish the conformational relationship and study the reaction mechanism.

## 2. Results and Discussion

### 2.1. Catalytic Performance

**Figure 1:**
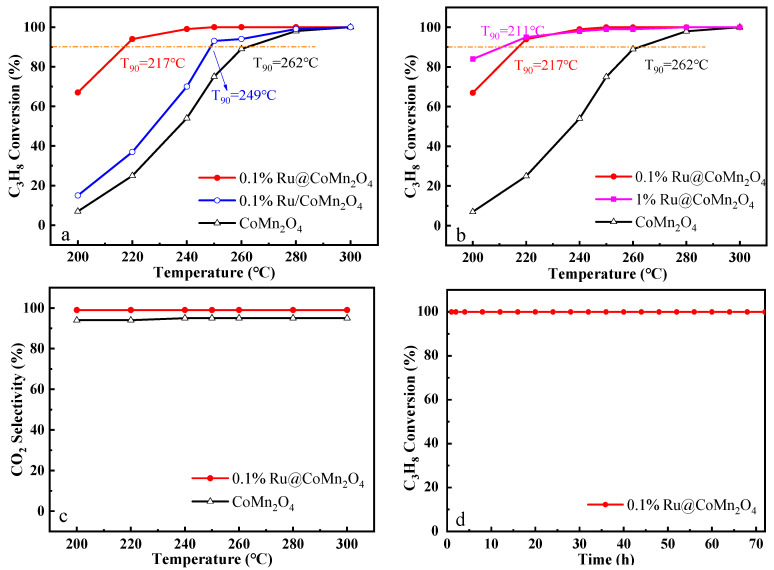
Effects of (**a**) different Ru loading methods and (**b**) loading content on catalytic activity of propane; (**c**) CO_2_ selectivity of catalysts (reaction conditions: 0.2 vol% C_3_H_8_, 10 vol% O_2_, N_2_ as balance gas, and GHSV = 60,000 mL g·cat^−1^ h^−1^); (**d**) stability of 0.1%Ru@CoMn_2_O_4_ catalyst (reaction conditions: 0.2 vol% C_3_H_8_, 10 vol% O_2_, N_2_ as balance gas, GHSV = 60,000 mL g·cat^−1^ h^−1^, and 250 °C).

The influence of Ru loading methods and Ru concentration on the catalytic combustion of propane were examined. It can be seen from Figure 1a that the introduction of Ru obviously improved the activity of propane catalytic combustion. The T_90_ (temperature under 90% propane conversion) of 0.1% Ru@CoMn_2_O_4_ and 0.1% Ru/CoMn_2_O_4_ was decreased by 45 °C and 13 °C, respectively, compared with that of CoMn_2_O_4_. Furthermore, the oxidation activity of the in situ doped 0.1% Ru@CoMn_2_O_4_ catalysts was significantly higher than that of the 0.1% Ru/CoMn_2_O_4_ catalyst obtained by the impregnation method. This may be because the in situ doped Ru was not only deposited on the surface of the catalyst but also dispersed inside the spinel, which resulted in higher activity and better stability. In a word, the Ru@CoMn_2_O_4_ catalyst displayed excellent catalytic activity, even better than some other noble-metal catalysts reported in recent years [27,28,29,30,31,32,33], indicating that the Ru@CoMn_2_O_4_ catalyst has potential for practical industrial applications in alkane removal. The catalyst activities in some of the relevant literature are shown in Table 1. It is worth noting that T_90_ of the Pt-Co_3_O_4_ NPs/PFs catalyst in ref. [30] was only 184 °C. This was due to its low GSHW (30,000 mL g·cat^−1^·h^−1^) and high Pt load (0.2 wt %). In addition, as shown in Figure 1b, the T_90_ of 0.1% Ru@CoMn_2_O_4_ was only 6 °C higher than that of 1% Ru@CoMn_2_O_4_. Considering the preparation of low noble-metal content, the subsequent physicochemical properties of the catalysts were investigated by using 0.1% Ru@CoMn_2_O_4_. The products of the propane oxidation were detected online with the DX4000 infrared analyzer (Gasmet, Vantaa, Finland). CO and other products were barely detected, at less than 1%. The CO_2_ selectivity of the catalysts is shown in Figure 1c. The CO_2_ selectivity of 0.1%Ru@CoMn_2_O_4_ approached 100%. It can be concluded that the 0.1%Ru@CoMn_2_O_4_ catalyst had excellent selectivity for propane oxidation. The stability of 0.1%Ru@CoMn_2_O_4_ is shown in Figure 1d. After 72 h of continuous reaction, C_3_H_8_ conversion was nearly constant, and no harmful by-products were detected, indicating that the 0.1%Ru@CoMn_2_O_4_ catalyst has high stability and potential for industrial applications in alkane removal.

### 2.2. Physical Characteristics

**Figure 2:**
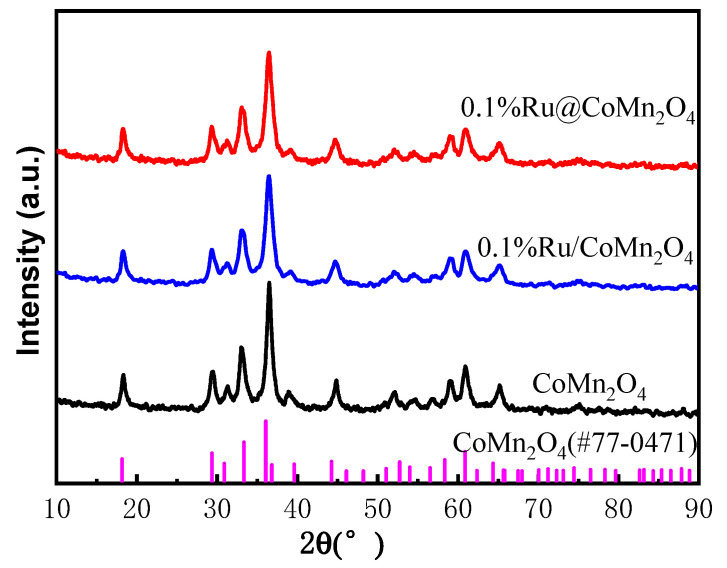
The XRD patterns of the catalysts.

XRD was used to determine the crystal structure of the spinel catalysts. The wide-angle XRD spectra of the samples are shown in Figure 2. It can be seen that the peak positions of 0.1%Ru@CoMn_2_O_4_, 0.1%Ru/CoMn_2_O_4_, and CoMn_2_O_4_ were basically the same as those of the standard card (JCPDS No. 77-0471) of CoMn_2_O_4_. These results suggest that the acetates of cobalt and manganese were decomposed at high temperatures into metal oxides and formed spinel catalysts by complete phase transitions, rather than simple mixtures of metal oxides. Specifically, the diffraction peaks situated at 18.2°, 29.2°, 31.2°, 33.0°, 36.3°, 44.8°, 59.0°, 60.9°, and 65.2° corresponded to the (101), (112), (200), (103), (211), (220), (321), (224), and (400) crystal planes of the CoMn_2_O_4_ spinel structure [34]. No diffraction peak of the Ru species was detected in 0.1%Ru@CoMn_2_O_4_ and 0.1%Ru/CoMn_2_O_4_, most likely due to the relatively low loading and uniform dispersal of Ru. In addition, the doping of Ru reduced the intensity of the diffraction peak, suggesting that the crystallinity decreased after Ru doping.

**Figure 3:**
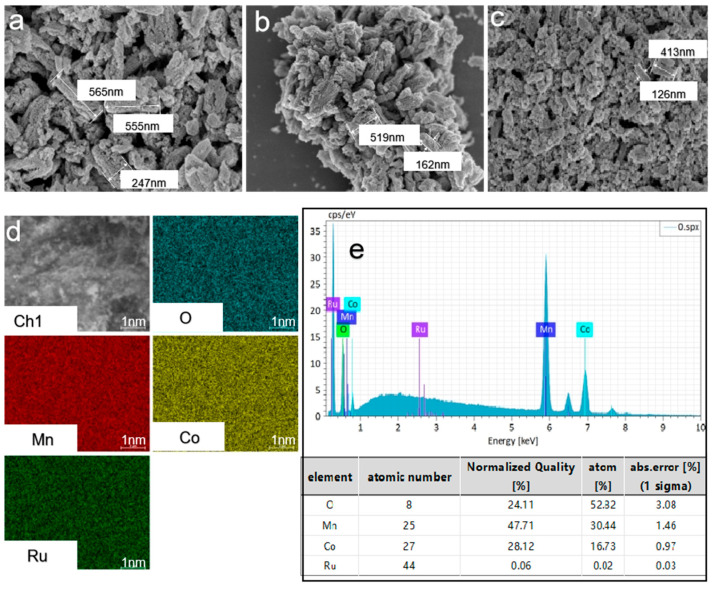
SEM images of (**a**) CoMn_2_O_4_, (**b**) 0.1%Ru@CoMn_2_O_4_, and (**c**) 0.1%Ru/CoMn_2_O_4_. (**d**) EDS energy spectrum data and (**e**) elemental line sweeps of 0.1%Ru@CoMn_2_O_4_.

SEM was conducted to examine the surface morphology of the catalysts, and the results are shown in Figure 3a–c. It can be seen that all of the catalysts were rod-like structures with lengths of about 500–600 nm, indicating that the doping of Ru did not significantly affect the morphology of the CoMn_2_O_4_ spinel. EDS mapping was conducted to further investigate the surface element distribution of 0.1%Ru@CoMn_2_O_4_. As shown in Figure 3d, Ru was uniformly dispersed on the CoMn_2_O_4_ spinel. In addition, the contents of O, Mn, Co, and Ru in 0.1%Ru@CoMn_2_O_4_ are illustrated in Figure 3e. The atomic ratio of Co/Mn was close to 1:2, which further verified the formation of the CoMn_2_O_4_ spinel. The content of Ru was 0.06%, slightly lower than the theoretical content.

**Figure 4:**
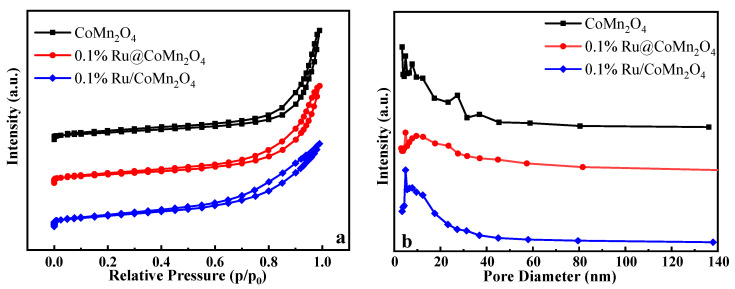
(**a**) N_2_ adsorption–desorption curves; (**b**) pore size distributions of the samples.

Figure 4 shows the nitrogen adsorption–desorption isotherms and the pore size distribution of the samples measured at 77 K. As can be seen from Figure 4, all of the samples exhibited typical type IV isotherms (IUPAC classification) with a separate H3 hysteresis loop, implying the formation of stacked pore structures. The abundant mesopores of the samples provided good diffusion conditions for the catalytic oxidation of propane [35]. The specific surface area, average pore size, and pore volume were calculated according to the classical BET and BJH models, and the structural parameters of the catalysts are summarized in Table 2. It can be seen that the surface area was obviously decreased after Ru doping, indicating that the introduction of the Ru species had a negative impact on the destruction of the textural structure. The surface area of 0.1%Ru@CoMn_2_O_4_ was smaller than that of 0.1%Ru/CoMn_2_O_4_, making it clear that the in situ introduction of Ru had a greater effect than the impregnation method. According to the activity evaluation results mentioned above, the physical properties were not the main factors affecting the activity of the catalyst.

### 2.3. Surface Chemical Properties

**Figure 5:**
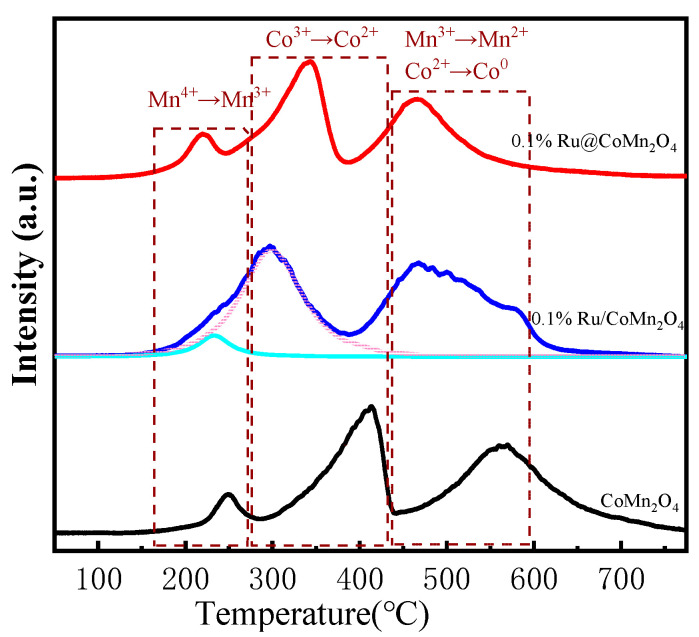
H_2_-TPR spectra of samples.

The redox capacity of the catalysts had a great impact on the catalytic activity for propane oxidation. The redox properties of the samples were investigated by H_2_-TPR, and the results are shown in Figure 5. Three reduction peaks can be observed in the H_2_-TPR curve of the CoMn_2_O_4_ catalyst. It contains four reduction processes. The peaks at 248 °C and 413 °C were attributed to the reduction of Mn^4+^→Mn^3+^ and of Co^3+^→Co^2+^, respectively. The peak at 565 °C contains two overlapped processes, including the reduction of Mn^3+^→Mn^2+^ and of Co^2+^→Co^0^ [36]. In contrast, the reduction peaks of 0.1% Ru@CoMn_2_O_4_ and 0.1%Ru/CoMn_2_O_4_ were obviously shifted in the low-temperature direction. Although the peak of Ru was not detected, the results sufficiently show that the doping of Ru significantly improved the low-temperature redox capacity of the catalyst. This may be owing to the stronger synergistic effect between the highly electronegative Ru and CoMn_2_O_4_; hence, the electrons were likely to migrate rapidly between them through the bridge oxygen structure. In addition, the reduction temperature of 0.1% Ru@CoMn_2_O_4_, especially for the shifting of Mn^4+^ to Mn^3+^, was lower than that of 0.1%Ru/CoMn_2_O_4_. Therefore, it can be concluded that the good low-temperature redox performance of 0.1% Ru@CoMn_2_O_4_ contributed to the acceleration of the catalytic oxidation cycle and improved propane oxidation activity to some extent.

**Figure 6:**
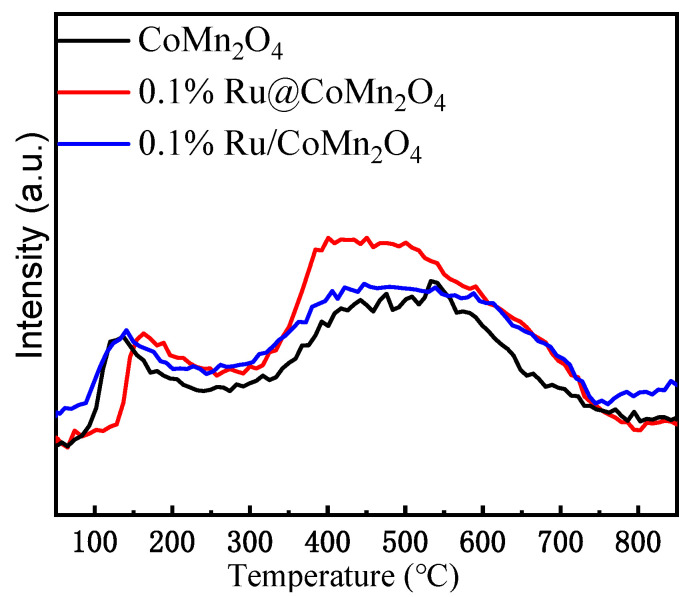
O_2_-TPD spectra of samples.

O_2_-TPD is an effective technique to study the desorption behavior of O_2_ on catalysts and is commonly used to evaluate the mobility and activation capacity of different oxygen species on catalysts. Typically, the capacity of oxygen desorption on the catalyst is O_2_(ad) > O_2_^−^(ad) > O^−^(ad) > O^2−^(lat) [37]. The adsorbed oxygen species (O_2_, O_2_^−^, and O^−^) are more readily desorbed than the lattice oxygen species [38]. Normally, oxygen species desorbed below 300 °C are physically/chemically adsorbed oxygen species on the catalyst surface, while oxygen species desorbed at higher temperatures (>300 °C) can be attributed to lattice oxygen. Based on the intensity of the oxygen desorption peaks (Figure 6), it can be seen that the content of lattice oxygen was 0.1%Ru@CoMn_2_O_4_ > 0.1%Ru/CoMn_2_O_4_ > CoMn_2_O_4_, and the temperature of the lattice oxygen desorption was shifted toward lower temperatures with the introduction of Ru, which suggests that Ru significantly enhanced the amount and mobility of lattice oxygen of the catalyst. It is possible that the highly electronegative Ru could promote the electron transfer from the cobalt–manganese spinel to Ru to form more lattice oxygen; at the same time, the doping of Ru caused the weakening of the bonding energies of Mn-O and Co-O, which increased the mobility of the lattice oxygens of CoMn_2_O_4_ and improved the catalytic oxidative performance of the lattice oxygen on propane [39,40,41,42]. In addition, the reduction temperature of 0.1% Ru@CoMn_2_O_4_, especially for the shifting of Mn^4+^ to Mn^3+^, was lower than that of 0.1%Ru/CoMn_2_O_4_. According to the results of H_2_-TPR and O_2_-TPD, it is concluded that the catalyst prepared by the in situ introduction of Ru had a better redox ability than that prepared by the impregnation method.

**Figure 7:**
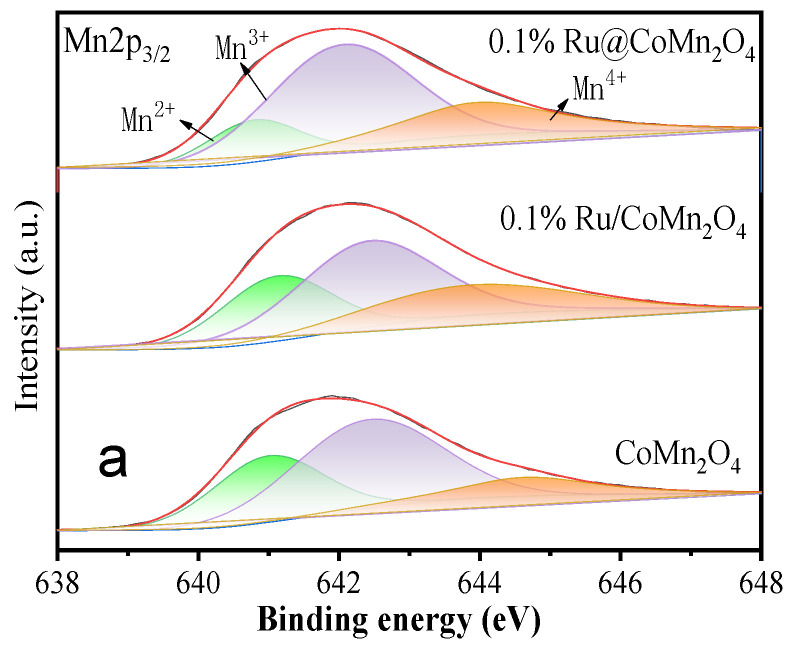

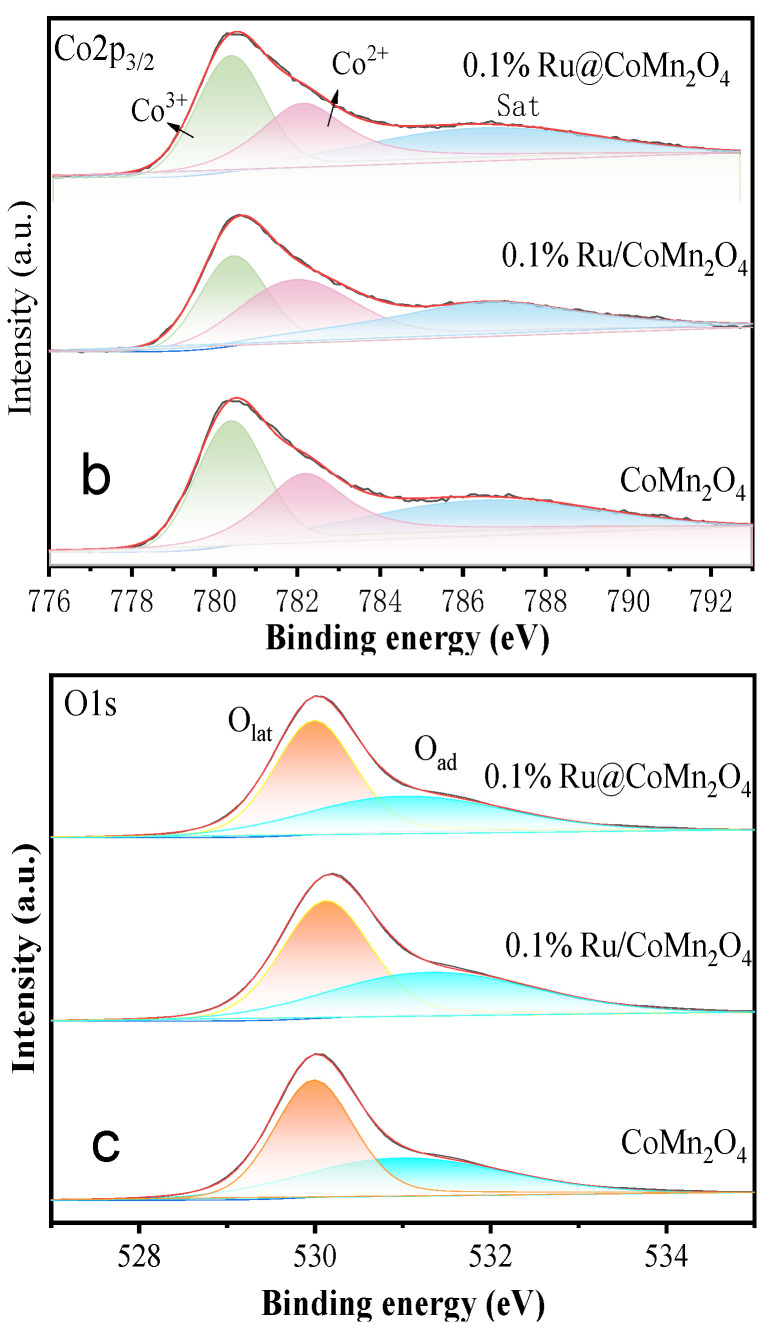
(**a**) Mn 2p, (**b**) Co 2p, and (**c**) O 1s XPS spectra of catalysts.

The surface state of the catalyst was analyzed by using the XPS technique to investigate the effect of cation substitution on the catalyst. Figure 7a demonstrates the high-resolution spectrum of Mn 2p_3/2_; the peaks at 640.8–641.0 eV, 642.0–642.4 eV, and 644.1–644.6 eV were attributed to Mn^2+^, Mn^3+^, and Mn^4+^, respectively [2,29,40]. The content of Mn^n+^ was calculated based on the fitted peak areas, and the results are summarized in Table 3. It can be observed that all of the samples were dominated by Mn^3+^. In addition, it is worth noting that the content of Mn^4+^ was significantly higher in the 0.1% Ru@CoMn_2_O_4_ catalyst compared with CoMn_2_O_4_. In the spinel structure, Mn preferentially occupied the octahedral coordination site as Mn^3+^. Partial Mn^3+^ cations were oxidized into Mn^4+^ in the octahedral sites due to the Jahn–Teller effect, which seriously distorted the localized lattice structure. As a result, the Mn-O bond energy became weaker, and the lattice oxygen easily participated in the reaction with propane [43,44]. It has been reported that the ratio of Mn^4+^/Mn is considered to have an important effect on the oxidation of VOCs, as the high-valence manganese species contribute to the oxidation of the catalysts [45]. Therefore, it was speculated that the doping of Ru, with the partial electron transfer from the lower electronegative Mn (X = 1.55) to the higher electronegative Ru (X = 2.20), elevated the valence of Mn at the octahedral sites, which contributed to the mobility of the lattice oxygen and facilitated the enhancement of the catalytic efficiency for propane.

Figure 7b shows the high-resolution spectrum of Co 2p_3/2_. The peaks at 780.4 eV, 782.2 eV, and 786.5 eV were attributed to Co^3+^, Co^2+^, and the strong satellite peak associated with Co^2+^, respectively [46]. Based on the spinel structure, it is hypothesized that Co was predominantly present on the tetrahedral structure and Mn on the octahedral sites. The metal ions at the center of the octahedral sites were directly opposite to O-2p, with a higher orbital overlap; hence, they possessed a higher oxygen binding capacity compared with the tetrahedral sites. Therefore, the Mn ions in the octahedral sites may be the main active sites for the propane oxidation reaction, while the redox cycle between Co^2+^ and Co^3+^ promoted the activation of propane oxidation.

The O 1s spectrum could be divided into two types of oxygen: lattice oxygen (O_lat_) at 530.0 eV and chemisorbed oxygen (O_ad_) at 531.5 eV [47]. The results of the split-peak fitting of the two oxygen species (Figure 7c and Table 3) showed that the O_lat_/O_ad_ ratios of CoMn_2_O_4_, 0.1%Ru/CoMn_2_O_4_, and 0.1%Ru@CoMn_2_O_4_ were 1.17, 1.27, and 1.36, respectively. Consistently with the previous conclusions of O_2_-TPD, it was regarded that the doping of Ru increased the amount of lattice oxygen. In addition, 0.1%Ru@CoMn_2_O_4_ possessed more lattice oxygen, not only loaded onto the spinel surface but also embedded in the crystal lattice.

### 2.4. Reaction Mechanism

**Figure 8:**
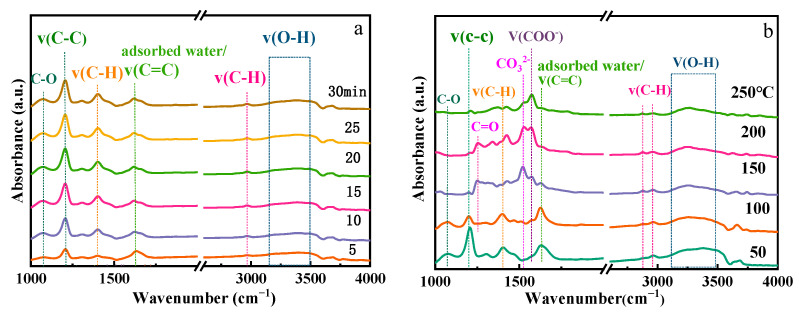
In situ DRIFT spectra of (**a**) C_3_H_8_ adsorption over 0.1%Ru@CoMn_2_O_4_ at 50 °C and (**b**) adsorbed species changes at different temperatures (reaction conditions: (**a**) 0.2 vol% C_3_H_8_/N_2_; (**b**) N_2_ atmosphere).

The intermediate species of the reaction between the adsorbed propane and lattice oxygen on the catalyst were monitored by in situ DRIFTS. Figure 8a shows the in situ DRIFT spectrum of propane adsorbed on the 0.1% Ru@CoMn_2_O_4_ catalyst at 50 °C. The peaks at 2967, 1402, and 1212 cm^−1^ were attributed to the C-H stretching vibration, C-H bending vibration, and C-C stretching vibration, respectively [27,48], indicating that propane was adsorbed on the catalyst surface. The peak at 1633 cm^−1^ was attributed to C=C stretching or adsorbed water [3,27,31,49,50]. The peak at 1054 cm^−1^ was attributed to the C-O stretching vibration of alcohols [27,29], which showed that propane could be partially oxidized to alcohols by lattice oxygen. The broad spectrum at 3200–3500 cm^−1^ was attributed to hydroxyl groups which might be from alcohol, carboxyl, or adsorbed water [31,32,51]. After 30 min of propane adsorption, C_3_H_8_ was cut off, and only N_2_ was left. The intermediate products were detected by increasing the temperature. As can be seen from Figure 8b, with the increase in the temperature, the peaks assigned to the C-O stretching vibration (1073 cm^−1^), C-C stretching vibration (1208 cm^−1^), C-H bending vibration (1402 cm^−1^), C-H stretching vibration (2872 cm^−1^, 2899 cm^−1^), and the C=C stretching (1622 cm^−1^) of propylene gradually weakened, while the vibrations of C=O (1249 cm^−1^), bidentate/uncoordinated CO_3_^2−^ (1515 cm^−1^), and acetate (1575 cm^−1^) appeared accordingly [27,29,31], implying that propane was further oxidized to carbonate and carboxylate species by lattice oxygen.

**Scheme 1:**
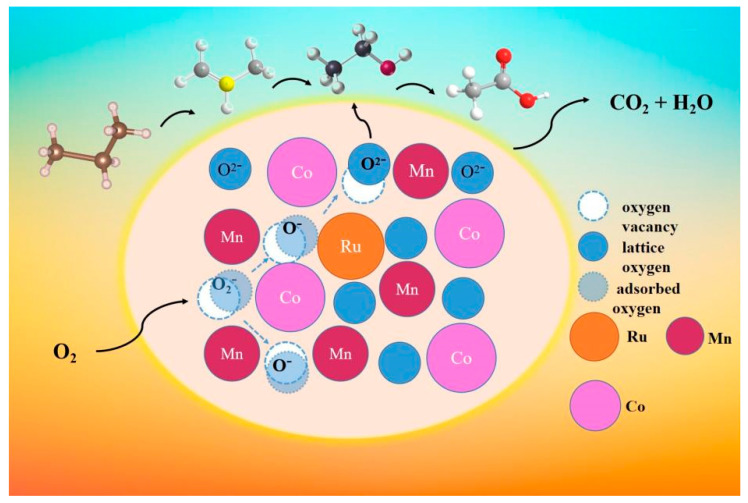
Proposed mechanism for propane oxidation over 0.1%Ru@CoMn_2_O_4_.

It has been reported that the oxidation of propane follows the Mars–van Krevelen (MvK) mechanism [27,52,53]; the process on 0.1%Ru@CoMn_2_O_4_ is hypothesized, as shown in Scheme 1. The highly electronegative Ru led to the transfer of electrons from Mn to Ru, which increased the valence state of Mn on the octahedral sites. Propane was adsorbed on the high-valence Mn cation (Mn^3+^, Mn^4+^) centers of the octahedral sites to form chemisorbed species, which reacted with the lattice oxygen associated with the high-valence Mn cations to obtain incomplete oxidation products (e.g., alcohols and carboxylic acids). At the same time, the high-valence Mn cations captured electrons and were reduced to low-valent Mn cations (e.g., Mn^2+^). Considering the redox couples of Mn^3+^/Mn^2+^ (E^θ^ = 1.54 V) and Co^3+^/Co^2+^ (E^θ^ = 1.92 V), Mn^2+^ tended to be oxidized by Co^3+^ [54], and lattice oxygen was transferred from the adjacent Co^3+^ center to the Mn^2+^ center to compensate for the loss of oxygen. Molecular oxygen could be adsorbed onto Co^2+^, where it was transformed into lattice oxygen. In this way, oxidation–reduction repeated indefinitely, allowing the reaction to continue until propane was completely oxidized to CO_2_ and H_2_O.

## 3. Experiment

### 3.1. Catalyst Preparation

Ru@CoMn_2_O_4_ was synthesized by following the sol–gel method. In a typical procedure, 4.90 g of manganese acetate (Aladdin, Shanghai, China, AR; 99.0%), 2.49 g of cobalt acetate (Aladdin, Shanghai, China, AR; 99.5%), and 0.0047 g of ruthenium trichloride (Macklin, Shanghai, China; 36.0–40.0%) were added to 150 mL of a mixed alcohol solution (ethanol:ethylene glycol = 3:1) (Macklin, Shanghai, China, AR; 99.5%) and stirred at 40 °C until completely dissolved to form solution A. A total of 4.5 g of Oxalic acid (Macklin, Shanghai, China, AR; 99.0%) was added to 50 mL of the above mixed alcohol solution and stirred until dissolved to form solution B. Solution B was added to solution A, and the mixture was stirred vigorously at 40 °C for 0.5 h. The precipitate was collected by centrifugation and dried at 80 °C for 24 h. The product was calcined at 300 °C for 3 h in an air atmosphere at a temperature rate of 5 °C/min and then kept at 240 °C for 1 h and at 80 °C for 1 h. Finally, the composite catalyst was obtained at room temperature, denoted as 0.1% Ru@CoMn_2_O_4_.

As a control sample, CoMn_2_O_4_ was prepared, referring to the method of 0.1% Ru@CoMn_2_O_4_, except for the absence of Ru. The other control sample (0.1% Ru/CoMn_2_O_4_) was prepared by using the impregnation method, impregnating the same mass fraction of Ru onto CoMn_2_O_4_.

### 3.2. Catalyst Activity Test

Catalytic activity was evaluated in a fixed-bed quartz reactor (id = 6 mm) at atmospheric pressure. The reaction gas consisted of 0.2% C_3_H_8_, 10% O_2_, and N_2_ as balance. A total of 0.1 g of the catalyst (40–60 mesh) was used in the reaction, and gas hourly space velocity (GHSV) was 60,000 mL g^−1^h^−1^. The temperature of the activity evaluation experiment ranged from 200 to 300 °C at the heating rate of 5 °C/min, and each temperature point was kept for 0.5 h. Stability tests were carried out at 250 °C under the same reaction conditions as the activity evaluation. The quantitative analysis of the reactants (C_3_H_8_) and products (CO_2_ and CO) was performed online by using the DX4000 infrared analyzer (Gasmet, Vantaa, Finland). C_3_H_8_ conversion was calculated by using the following equation:

$$X_{C3H8}=\frac{C_{in}-C_{out}}{C_{in}}\times 100\%$$

where *C_in_* and *C_out_* represent the C_3_H_8_ concentrations assigned to the inlet and outlet, respectively.

### 3.3. Material Characterization

The structures were determined by using a Bruker D8 Advance X-ray diffractometer (XRD) (Bruker, Ettlingen, Germany) with Cu Ka radiation (40 mA, 40 kV). N_2_ adsorption–desorption isotherms were obtained by using a Quantachrome Auto Sorb iQ-MP (Quantachrome, Boynton Beach, FL, USA). The BJH model was used to evaluate the pore size distribution, and the BET multi-molecular adsorption model was used to evaluate the surface area. The surface morphology of the samples was examined by JSM-7001F field emission SEM (JEOL Ltd., Tokyo, Japan), while the elemental distribution was measured by EDS. H_2_-TPR and O_2_-TPD were conducted on a Quantachrome ChemStar (Quantachrome, Boynton Beach, FL, USA)with a thermal conductivity detector (TCD) to detect the signals. XPS data were collected by using AXIS ULTRA DLD photoelectron spectroscopy (Shimadzu, Kyoto, Japan) with Al Kα X-ray radiation (1486.6 eV).

In situ DRIFTS was performed with Tensor 27 (Bruker, Ettlingen, Germany). First, degassing was carried out at 300 °C for 1 h by using N_2_ as the desorption gas. After cooling, 2000 ppm C_3_H_8_/N_2_ was introduced for adsorption (25 mL·min^−1^) till saturation to detect the process of propane adsorption. Then, C_3_H_8_ was cut off, and the temperature was increased at the heating rate of 5 °C/min to detect the changes in the surface species of the catalyst in N_2_ atmosphere. The temperature ranged from 50 to 250 °C, and each temperature point was kept for 0.5 h till stabilization. Infrared spectral data of the samples were acquired by accumulating 64 scans at a resolution of 8 nm^−1^. All of the data were subjected to background subtraction.

## 4. Conclusions

In conclusion, Ru doping has brought a significant improvement in the propane catalytic performance of CoMn_2_O_4_ spinel catalysts. Specifically, 0.1%Ru@CoMn_2_O_4_ displayed excellent catalytic activity and stability. The T90 of 0.1%Ru@CoMn_2_O_4_ was 217 °C, lower than that of CoMn_2_O_4_. H_2_-TPR, O_2_-TPD, and XPS results show that the electronic structure of CoMn_2_O_4_ was obviously modulated by the charge redistribution with the introduction of Ru. More high-valence Mn^4+^ species were generated on the surface, and lattice oxygen species were easier to mobilize due to the charge transfer and synergy between Mn and Co, facilitating the adsorption and activation capacity for propane. In addition, in situ DRIFTS revealed another path with olefin species as the intermediates on 0.1%Ru@CoMn_2_O_4_ surface, which is conducive to a clearer understanding of the propane oxidation path. This work uncovers the electronic regulation effect of the Ru species doped in CoMn_2_O_4_ on C_3_H_8_ oxidation and provides guidelines for light-alkane removal applications. Overall, it provides a design strategy for novel catalysts with dual-active-component catalysts and extends the application of these catalysts in the important field of VOC elimination.

## Figures and Tables

**Table 1:** Comparison of catalytic results for 0.1%Ru@CoMn_2_O_4_ catalyst with those reported in the literature.

Catalyst	Reaction Conditions	T90 (°C)	Ref.
NiMn_2_O_4_	0·05% C_3_H_8_, 5% O_2_, GHSV = 30,000 mL g·cat^−1^·h^−1^	232	[27]
Ag/MnCeO_x−7_	0·25%C_3_H_8_, 21%O_2_, GHSV = 30,000 mL g·cat^−1^·h^−1^	242	[28]
Pd-Mn_3_O_4_	0·25%C_3_H_8_, and balance air, at 30,000 mL g·cat^−1^·h^−1^	240	[29]
Pt-Co_3_O_4_ NPs/PFs	0·25%C_3_H_8_, and balance air, at 30,000 mL g·cat^−1^·h^−1^	184	[30]
MnxCr_3−x_O_4_	0·25%C_3_H_8_, and balance air, at 30,000 mL g·cat^−1^·h^−1^	264	[31]
ZnCoMn spinel	800 ppm C_3_H_8_, and balance air, at 30,000 mL g·cat^−1^·h^−1^	360	[32]
Co_3_O_4_/SmMn_2_O_5_	0·2%C_3_H_8_, 2%O_2_, 5% H_2_O, at 30,000 mL g·cat^−1^·h^−1^	247	[33]
0.1%Ru@CoMn_2_O_4_	0·2% C_3_H_8_, 10% O_2_, GHSV = 60,000 mL g·cat^−1^·h^−1^	217	This work

**Table 2:** The porous structural parameters and T_90_ of the samples.

Catalyst	S_BET_ (m^2^/g)	Average Pore Size (nm)	Pore Volume (cm^3^/g)
CoMn_2_O_4_	132.57	14.13	0.47
0.1%Ru/CoMn_2_O_4_	106.27	11.54	0.31
0.1%Ru@CoMn_2_O_4_	82.97	17.22	0.36

**Table 3:** XPS results of surface Co, Mn, and O.

Catalyst	Mn^2+^/Mn	Mn^3+^/Mn	Mn^4+^/Mn	Co^2+^/Co	Co^3+^/Co	O_lat_/O_total_	O_ad_/O_total_
CoMn_2_O_4_	0.31	0.54	0.15	0.47	0.53	0.54	0.46
0.1%Ru/CoMn_2_O_4_	0.27	0.49	0.24	0.54	0.46	0.56	0.44
0.1%Ru@CoMn_2_O_4_	0.15	0.58	0.27	0.48	0.52	0.59	0.41

## Data Availability

The data presented in this study are available in article.
